# Medical education in difficult circumstances: analysis of the experience of clinical medical students following the new innovative medical curriculum in Aksum, rural Ethiopia

**DOI:** 10.1186/s12909-018-1199-x

**Published:** 2018-05-31

**Authors:** C. Morgan, M. Teshome, T. Crocker-Buque, R. Bhudia, K. Singh

**Affiliations:** 1 Barts and the London School of Medicine and Dentistry, Queen Mary, University of London, EC1M 6BQ, London, UK; 2 Faculty of Health Sciences, Aksum University, Aksum, Ethiopia

**Keywords:** Curriculum, Medical education, Clinical experience, Africa

## Abstract

**Background:**

In 2012, 12 medical schools were opened in Ethiopia to tackle the significant shortage of doctors. This included Aksum School of Medicine situated in Aksum, a rural town in Northern Ethiopia. The new Innovative Medical Curriculum (NIMC) is a four-year programme designed by the Ethiopian Federal Ministries of Health and Education. The curriculum is designed to train biomedical science graduates to become doctors in 4 years, with a focus on the healthcare needs of rural people living in poverty.

**Methods:**

This research was conducted at Aksum School of Medicine and included two hospitals (Aksum Referral Hospital and St Mary’s District Hospital). This study focused on medical students during their clinical years across multiple specialities (61 Clerkship 1 students and 13 Clerkship 2 students). We used primarily qualitative research methods supplemented with quantitative measures. There were 3 stages of data collection over a 1 month period, this included qualitative group interviews, direct observation of students in a clinical setting and direct observation of skills sessions followed by a questionnaire on the sessions. We analysed the data by reconstructing the student experience and comparing it with the NIMC.

**Results:**

The proposed typical week set out in the NIMC tended to differ from the real clinical experience of these students. Through qualitative group interview and direct observation of teaching, the main theme that was consistent throughout was the lack of doctors with specialist postgraduate training. Clinical need often took priority over education. However, students enjoyed taking early responsibility and gaining practical experience. Through direct observation of skills sessions and short questionnaires, these sessions were highly valuable to the students and they felt confident in carrying out the taught procedures in the future.

**Conclusions:**

The combination of poorly resourced hospitals and lack of specialist doctors provides a challenging environment for medical students to learn. However, it is a unique clinical experience that is rarely seen in developed countries and facilitates the acquirement of skills from an early stage. Supervision and specialist input is fundamental in enabling students to learn and this is a key area that was lacking in the students’ clinical experience.

## Background

Ethiopia is a country with a severe shortage of doctors especially in the rural areas [1]. According to the World Health Organization (WHO), in 2010 the doctor to patient ratio in Ethiopia was 1:42,706, which is among the lowest in sub-Saharan Africa [1]. The WHO established a critical threshold of 23 doctors, nurses and midwives per 10,000 population as a minimum to enable the delivery of essential maternal and child health services, yet they found this figure to be 3 per 10,000 in Ethiopia [2]. Furthermore, it is estimated that 43% of doctors work in Addis Ababa (the capital of Ethiopia) despite only 5% of the population living there [1]. The severe shortage of doctors in Ethiopia could be attributed to several reasons including the emigration of qualified doctors, limited number of medical schools, and difficulty in accessing education [3].

Aksum is a town in Tigray, Northern Ethiopia with an estimated population of 66,800 people [4]. Aksum University Faculty of Health Sciences was opened in 2012 along with 11 other medical schools to tackle the significant shortage of doctors in Ethiopia. The New Innovative Medical Curriculum (NIMC) is a four-year programme designed by the Ethiopian Federal Ministries of Health and Education [5], which has been in place in Aksum since 2012. There have been 4 years of clinical students on the course, with the first cohort of students graduating in 2016. The curriculum is designed to train biomedical science graduates to become doctors in 4 years, with a focus on the healthcare needs of rural people living in poverty.

**Fig. 1:**
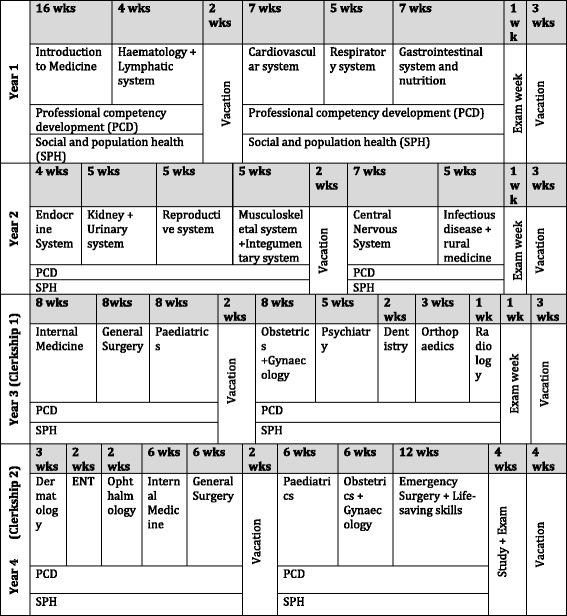
An overall summary of the curriculum taken and adapted from the New Innovative Curriculum [NIMC] document

The four-year curriculum is split into two pre-clinical years and two clinical or clerkship years, known as Clerkship 1 (C1) and Clerkship 2 (C2) (see Fig. 1). After these 4 years, the students will then undertake an internship year. Upon completion of each academic year there are examinations and at the end of C2, the students sit their final qualifying examination.

There is known to be variation in clinical teaching experienced by medical students and that this often differs from the curriculum intended to be delivered [6, 7]. However, evidence has shown the benefits of implementing a monitoring system that allows comparisons to be made across specialities and hospitals. This also allows the identification of areas that are lacking in the student’s clinical experience [8–10]. To our knowledge this is the first paper examining the implementation of the NIMC in Ethiopia.

## Methods

The aim of this research is to identify and describe the real-world experience of the clinical students following the NIMC at Aksum and compare this to the proposed curriculum experiences and outcomes.

### Research design

We evaluated the experience of clinical students using qualitative methods supplemented with quantitative measures. The research was conducted at Aksum School of Medicine and included two hospitals (Aksum Referral Hospital and St Mary’s District Hospital). The research included 61 C1 students and 13 C2 students.

There were 3 stages of data collection, which we completed over a 1 month period:

1. Qualitative group interviews
2. Direct observation of students in a clinical setting
3. Direct observation of skills sessions followed by a student questionnaire on the sessions

We completed Stages 1 and 2 over a 2-week period. Stage 1 consisted of eight structured group interviews, involving 2–4 participants per interview. We conducted the interviews using a convenience sample of available students in the clinical area. We used this method as it allowed the students to decide if they wished to participate, any students who wanted to participate were included in the study. It was not possible to pre-arrange interviews with the students due to network and power outages which sometimes lasted multiple days making communication and organisation of meetings very difficult.

We used an interview guide that covered the students’ experience in Ophthalmology, Dermatology, Obstetrics and Gynaecology and Surgery. CM and MT developed the guide after discussion with senior faculty members from Aksum University and review of the NIMC. We asked the students to describe their current clinical experience, including details of their attachment and their average day. We also asked what they felt worked well on their attachment and areas that could be improved. The lead researcher (CM) attended both hospitals (Aksum Referral Hospital and St Mary’s District Hospital) alternating sites over a two-week period to carry out the structured group interviews alongside a General Practitioner from Aksum (MT). CM and MT conducted these interviews together in the morning after locating the medical students in the relevant departments within the hospital. The morning was the time when the highest number of students were present. The lead researcher (CM) collected the data through note taking. The Aksum GP was known to the students which enabled a strong rapport and trust to be built during the interview.

We obtained verbal consent prior to interviewing the students and all data was anonymised to maintain confidentiality. We retained a written register of the students who verbally consented. We offered the students the choice of participating and we explained that it would not affect their training in any way if they chose not to participate. We also obtained verbal consent from the consultant in charge of each department prior to direct observation and interviews. All interviews and teaching sessions were conducted in English, which is the same language the students are taught in. In Stage 2, CM observed a selection of students, based on convenience sampling, in the clinical environment. This involved both hospital wards and outpatient clinics. CM made observational notes to compare with the described student experience in the NIMC. We obtained ethical approval from both Aksum University and Queen Mary University of London. All researchers had received training in collecting qualitative data.

Two medical students (RB & KS) carried out Stage 3 of data collection. This involved direct observation of skills sessions using a structured observation teaching form, followed by an evaluation of the students’ experience using a cross-sectional survey. We anonymised the survey to maintain the students’ confidentiality. We (RB & KS) collected data by observational note keeping. A debrief was undertaken after each session. The researchers (RB & KS) then compared the notes, collaborated findings and any differences were discussed. Both medical students (RB & KS) were present at all skill sessions. The survey consisted of the following 5 questions (based on a Likert-type scale):

1.  How useful did you find the teaching?
2.  Did you feel the teaching was right for your level?
3.  How confident would you be in using the skills you learnt in the session?
4.  Was the session well organised?
5.  What other aspects of this topic would you like to see included?

### Data analysis

The lead researcher (CM) collated the observational notes from the three researchers who had taken notes (CM, RB & KS) into a written document. This data included qualitative group interviews, direct observation of teaching and direct observation of skills sessions. The lead researcher (CM) developed deductive codes which were derived from the interview guide, these included ‘positive experience’, ‘areas for improvement’ and ‘structure’. CM then developed inductive codes from further reading and analysis of the data. These included ‘supervision’, ‘teaching’, ‘level of responsibility’ and ‘workplace dynamics’. Coding of the data allowed common themes to be identified. The lead researcher (CM) then placed the data into different categories, consisting of headings and subheadings which involved the year and speciality of the medical students. CM then mapped the data to the NIMC. We categorised the data in this way as it allowed conclusions to be drawn and recommendations to be made to the faculty leadership and individual departments. Using the Likert-type scale, researchers RB and KS collated the results from the cross-sectional survey into a simple bar chart. An experienced qualitative researcher (TCB) checked the data analysis and coding framework. This helped to ensure reliability in the data analysis and credibility of the coding. TCB identified any discrepancies in the coding and revisions were made.

## Results

### Stage 1 (qualitative group interviews) and stage 2 (direct observation of teaching)

The main theme that was consistent throughout was the lack of doctors with specialist postgraduate training. Students enjoyed being taught by specialists when available, however, there were different levels of engagement across the different specialities. This was often due to clinical need taking priority over education and specialists having to leave to attend emergencies, particularly in the case of Surgery and Obstetrics. There was often poor access to basic teaching materials such as ophthalmoscopes and specialist textbooks. However, students enjoyed taking early responsibility and gaining practical experience, for example delivering babies and performing minor surgical procedures. The proposed typical week set out in the NIMC for C1 and C2 students (see Table 1) tended to differ from the real clinical experience of these students.

**Table 1:** A proposed typical week in Clerkship 1 and 2 taken from the NIMC

Time	Monday	Tuesday	Wednesday	Thursday	Friday
8 am – 10 am	Lecture (C1) / Morning session (C2)	Lecture (C1) / Morning session (C2)	Social and Population Health	Lecture (C1)/ Morning session (C2)	Primary Health / Community attachment
10 am-12:30 pm	Bedside teaching	Inpatient/ outpatient care	Inpatient/ outpatient care	Bedside teaching	
12:30 pm- 2 pm	Lunch	Lunch	Lunch	Lunch	Lunch
2 pm–4 pm	Inpatient/ outpatient care	PCD	Inpatient/ outpatient care	Inpatient/ outpatient care	Whole group session seminar/journal club/grand round
4 pm-5:30 pm		Inpatient/ outpatient care			

#### Clerkship 1

Sixty-one medical students were studying Dermatology (30) and Ophthalmology (31) at Aksum Referral Hospital. The students were split into two groups and then into further subgroups of approximately 8, each student spent 3 weeks on Ophthalmology and 3 weeks on Dermatology. Aksum Referral Hospital had just recently opened and all clinical exposure was outpatient based. There was one specialist Ophthalmologist and one specialist Dermatologist. An average day would involve teaching by the specialist in the morning, via a PowerPoint presentation that was then followed by a clinic. Groups had to have at least 1 day per week away from the clinical environment due to there being only one specialist in each department and 61 students.

##### Ophthalmology

From direct observation, the ophthalmology clinic consisted of the ophthalmologist, specialist nurse and medical students. There was one ophthalmoscope in the department used by the specialist and a slit lamp; the students used a torch and goggles to examine the eyes. Jointly the students would take a very brief history from the patient with the nurse. There appeared to be a good relationship between the specialist nurse and the students, allowing the students time to examine the patients. The students would then observe the ophthalmologist who at times gave teaching. The relationship between the students and ophthalmologist appeared strained, primarily due to the clinical work load. From observation, it was clear that the students were being exposed to a wide variety of ocular pathology with advanced clinical signs. During the interviews the students spoke about the lack of hard copy Ophthalmology textbooks in the library and their reliance on a limited Internet connection for resources. They also spoke about their desire to learn how to use equipment such as an ophthalmoscope.

##### Dermatology

The dermatology clinic comprised of the dermatologist and the medical students. From direct observation, approximately 8 students stood around the patient and the students were asked to take a history from the patient, formulate differential diagnoses and consider appropriate investigations. Possible management plans were then discussed with the students. From observation, the dermatologist appeared highly motivated with a passion for teaching. The dermatologist ensured all students were involved in the teaching and reading materials were given prior to the clinic. Much like Ophthalmology, when interviewing the students, they spoke about limited access to textbooks and difficulty accessing online resources. However, they enjoyed seeing a wide variety of dermatological conditions and were very positive in regards to the dermatologist.

#### Clerkship 2

Thirteen students were on the Emergency Surgery and Lifesaving skills module at St Mary’s District Hospital Aksum. This is a 12-week module made up of 6 weeks of Surgery (6 students) and 6 weeks of Obstetrics and Gynaecology (7 students) and is the final module before they sit their final qualifying exam. The students were split into two groups and rotated after 6 weeks.

##### Surgery

There was one general surgeon covering the entire hospital and the different surgical subspecialties. The students’ surgical rotation was split into 2 weeks of theatre and 4 weeks of ward work. Theatre involved assisting in major surgery, involving all sub surgical sub-specialities, which included plastics, vascular, orthopaedics and paediatric surgery. They also undertook minor procedures unsupervised, such as draining abscesses. From direct observation on the wards the students would clerk patients, prescribe medications, consent patients for surgery and manage uncomplicated patients. From group interview the students sometimes felt overwhelmed by the responsibility given to them and the number of unwell patients. They also found the working conditions hard, these included no running water in the hospital and lack of basic medical supplies. However, as they were in their final year, they felt this responsibility and experience was important in preparing them for their internship year.

##### Obstetrics and Gynaecology

The responsibility given to these final year medical students was significant. There was one specialist across the entire department. The students were running their own clinics and admitting, managing, prescribing medications and discharging patients (see Table 2). They were also performing surgical procedures such as manual vacuum aspiration without supervision. The students were using forceps and ventouse devices supervised by midwives as well as delivering babies unsupervised. They were also managing emergencies such as postpartum haemorrhage, which was observed during the data collection. There appeared to be a good relationship between the students and the specialist, with morning meetings approximately three times a week to discuss current and difficult cases. The students also appeared to be well supported by the midwives. During group interview the students commented that often the specialist had to leave the hospital due to obstetric emergencies in other rural health centres where the patients were unable to access transport to the hospital. However, the time spent with the specialist, medical students found very valuable to their learning.

**Table 2:** Typical day for a final year medical student on Obstetrics and Gynaecology reconstructed from direct observation and student interview

Time	Typical day for a final year medical student on Obstetrics and Gynaecology Rotation
7:30 am	Arrive at St Mary’s Hospital via mini-bus.
8 am – 9 am	Morning meeting led by the specialist [twice a week] to discuss complicated patients and deliveries.
10 am–11 am	Ward round – led by specialist [twice a week], other times carried out by masters students or the medical students without supervision.
11 am – 12 pm	The 6 students would split off – 2 would run the outpatient clinic, 2 would be on the general ward, 2 would be on labour ward with little or no supervision
12 pm–1 pm	Mini-bus would take the students back to their accommodation for lunch.
1 pm–5 pm	The students would continue in their designated areas. The students on labour ward were also able to go to theatre. Twice a week the students would have a tutorial where they would take turns at presenting a topic to the other students and specialist.
5 pm–6 pm	The mini-bus would take the students back to their accommodation.
6 pm-8 am	Each student would work one night and one Saturday or Sunday per week – known as ‘duty’. They would then stay at the hospital the following day after a night duty.

### Stage 3: Direct observation of skills sessions followed by a short questionnaire

The skills sessions consisted of 60-min-long tutorials, delivered by two senior clinicians from the obstetrics department, to groups of 15 students. The sessions were conducted in a well-equipped skills lab. The content of the session would be oriented to lecture based teaching that had been delivered by residents in the same week. For example, on a week discussing termination of pregnancy, the skills session was to familiarise students with manual vacuum aspiration. These topics would also be selected based on feedback from previous students who had struggled with this area in their assessments. The initial skills session would act more as an interactive tutorial, establishing the students’ prior knowledge and introducing them to the equipment generally, before being followed by a supervised practice session.

**Fig. 2:**
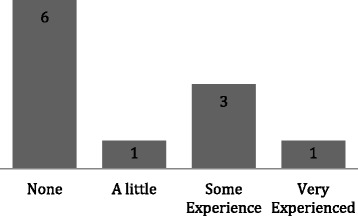
A bar chart showing the students previous experience of the skills being taught (*n* = 11)

**Fig. 3:**
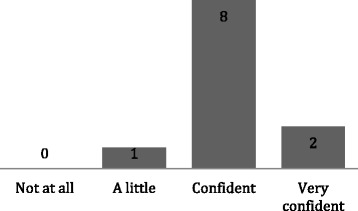
A bar chart showing the confidence of students using the skills learnt in the session (n = 11)

These skills sessions were well received by the students who mostly had little or no prior experience of the procedure being taught (Fig. 2). The interactive nature of each session, in addition to students receiving feedback and being supervised while practicing resulted in 91% of students reporting feeling either confident or very confident to carry out the procedure in the future (Fig. 3). Four students did not respond to the questionnaire.

Medical student comments were also broadly positive about the session stating that it was “necessary” and should be “continued” while also seeking further opportunity to practice, either in the skills lab or on patients.

## Discussion

The combination of poorly resourced hospitals and lack of specialist doctors provides a difficult environment for medical students to learn. A shortage of specialist doctors is a huge problem across Ethiopia, especially in rural areas such as Aksum. Supervision is fundamental in enabling students to learn and progress into safe clinicians [11] and this is a key area that was lacking in the students’ clinical experience. This highlights the urgent need for more specialist doctors or trainee specialists in areas such as Aksum, alongside stronger guidance on medical students’ duties and responsibilities. Nevertheless, it should be remembered that the NIMC was put in place to address this shortage of doctors, therefore there is not a quick fix to this problem but a longer-term strategy in place. A recent study at a medical school in Tanzania has identified the need for the curriculum to clearly specify the level of competence required for performing certain procedures [12]. This ensures the students are clinically competent in certain procedures when graduating medical school, which is something that could be implemented into the NIMC.

Furthermore, Fig. 1 clearly demonstrates the summary of the NIMC for each year. However, what should be noted is that Dermatology and Ophthalmology are specialties that are timetabled for C2 students. However, during the data collection period, the C1 students were on Dermatology and Ophthalmology attachments. The faculty of medicine in Aksum explained this change was due to the difficulty in recruiting specialist doctors to deliver parts of the curriculum, for example in Radiology or ENT. Therefore, they had to reach a compromise and since there was a specialist Dermatologist and Ophthalmologist available, they decided to deliver these areas during C1 instead of C2. This highlights the difficulty in delivering a curriculum in a developing country and the challenges that need to be overcome. However, it also demonstrates the forward thinking of the Aksum leaders in their ability to quickly adapt to the circumstances and to deliver successful modules in Dermatology and Ophthalmology. A study carried out in Kenya evaluating the quality and relevance of curriculum development in health training, found that there are major gaps in the quality and adequacy of the training [13]. The study also highlighted the need for a national standard framework to guide curriculum review.

A common theme that should be discussed is the different levels of engagement from the specialist doctors. Each department had only one specialist which placed enormous strain on these doctors. St Mary’s, a 150 bed district general hospital in Aksum is estimated to serve 1.5 million people. The burden of disease in Ethiopia measured in terms of premature death is estimated at 350 disability adjusted life years lost per 1000 population, which is the highest in Sub-Saharan Africa [14]. The combination of the disease burden and one specialist per department often leads to specialists being called away to emergencies. This can lead to students being left on the wards unsupervised potentially placing themselves in difficult situations. However, overall the students seemed to have a very positive approach to these circumstances and were happy to take on the added responsibility and adapt to meet the patient demand. Other healthcare professionals such as specialist nurses and midwifes were also very supportive of the students, demonstrating a team approach in providing the best patient care possible with the limited resources available.

High levels of responsibility and a huge disease burden can be advantageous to a medical student’s education. Exposure to tropical diseases such as Leishmaniasis and patients consistently presenting at extremely advanced stages of disease is a unique experience that is rarely seen in developed countries. This allows Aksum medical students to continue to develop their history and examination skills and formulate diagnoses without becoming dependent on investigations. The Aksum students enjoyed gaining practical experience which involved delivering babies and performing minor surgical operations. A study carried out in Tanzania found that clinicians felt clerkship students should be performing procedures such as a lumbar puncture under minimal supervision [12]. Allowing the medical students to develop these practical skills and gain ‘hands-on’ experience is important for when they take on further responsibilities as qualified doctors.

It does appear that the curriculum undergoes some degree of tailoring to knowledge gaps identified in assessment, as evidenced by the skills sessions. The fact that these sessions were engaging, interactive and resulted in the students having a high level of confidence in their ability to perform the taught practical skills, are all examples of good educational practice. The development of confidence in practical procedures is hugely important for when these skills are transferred into a clinical setting, especially if the students are performing skills with limited supervision. Students’ positive comments in feedback further illustrates the value of this practice. Further collection of feedback and tailoring of the curriculum to the students’ needs should be encouraged [15].

A limitation of this study is the fact that convenience sampling was used, which may not be representative of the whole cohort of students. Only 13 C2 students were involved in this study due to the remaining C2 students being placed at hospitals outside of Aksum; these hospitals were not accessible by public transport. To ensure the data is fully representative further research could be conducted in these hospitals. Further research could also explore different specialties that were not covered in this study. Another limitation to the data collection is that the interviews were not directly recorded and transcribed due to the lack of available equipment in Ethiopia.

Access to basic teaching materials for specialities such as Ophthalmology and Dermatology are very important in the students’ learning and their absence was a common theme highlighted by the students. This was the first time that Aksum Referral Hospital had Dermatology and Ophthalmology teaching clinics for the students as the hospital had recently opened and these departments were the first to become operational. The medical school recruited the patients for the clinic by organising a campaign in the week prior to the clinic. This involved an individual with a ‘megaphone’ driving up and down the main road in Aksum inviting anyone who had any eye or skin problems to come to the hospital. This again demonstrates the creativity of the medical school in organising and delivering the curriculum. As more departments open this will provide invaluable learning opportunities across different specialities for the medical students. It is important that Ophthalmology and Dermatology resources including textbooks are available for the students to help consolidate their learning. Investing in equipment such as ophthalmoscopes and teaching the students how to use them will provide them with important skills that they can apply in hospitals throughout Ethiopia [16].

A recent study looking at how medical students contribute to healthcare in Sub-Saharan Africa showed students are valuable resources in the health facilities [17]. They had a positive effect on workload, quality of care and working environment. This also appeared evident from observation of the students in Aksum, highlighting the positive impact that students can have in rural hospitals. It is however important to have the resources to support these students to ensure they further their knowledge and develop into competent doctors.

## Conclusion

Clinical medical students at Aksum University have high levels of experience, expertise and responsibility. However, there is a need for more senior supervision across the field to maximise the students’ clinical experience and to optimise patient care. The implementation of the NIMC is an important area that should continue to be reviewed regularly, not only in Aksum but across all medical schools throughout Ethiopia.

## Abbreviations

C1: Clerkship 1
C2: Clerkship 2
NIMC: New Innovative Medical Curriculum
PCD: Professional competency development
SPH: Social and population health
WHO: World Health Organization
